# Downregulation of the Long Non-Coding RNA *MDL1AS* Alters Metabolism, Differentiation, and Radiosensitivity in NTERA2 and SH-SY5Y Cells

**DOI:** 10.3390/cancers18060928

**Published:** 2026-03-12

**Authors:** Adrián Casas-Benito, Pablo Garrido, Alfredo Martínez

**Affiliations:** Angiogenesis Group, Oncology Area, Center for Biomedical Research of La Rioja (CIBIR), 26006 Logroño, Spain; acasas@riojasalud.es (A.C.-B.); pgarridor@riojasalud.es (P.G.)

**Keywords:** *MDL1AS*, NTERA2, SH-SY5Y, differentiation, radiation sensitivity, metabolism, glycolysis, DDR, Lamin B1, PAI-1

## Abstract

**Background/Objectives**: Non-coding RNAs provide new chances of targeting multiple oncogenic pathways. Many long non-coding RNAs (lncRNAs) are being characterized as relevant in cancer initiation, progression, and recurrence. *Mitochondrial D-loop 1 antisense lncRNA (MDL1AS)* is a novel lncRNA that might be important in cancer development, so the aim of this project was to understand its function in differently differentiated cancer cells. **Methods**: The effects of *MDL1AS* downregulation on the cellular behavior of NTERA2 and SH-SY5Y cell lines were studied. **Results**: *MDL1AS* reduction inhibited oxidative phosphorylation in NTERA2 cells and induced neuritic differentiation in SH-SY5Y cells. This downregulation also produced a strong DNA damage response (DDR) and an increased apoptotic signature by RNAseq analysis, and decreased proliferation in both cell lines. It also decreased radiosensitivity in NTERA2 cells but not in SH-SY5Y. **Conclusions**: These results suggest that *MDL1AS* reduction can modulate radiosensitivity, metabolism, and differentiation in a cell type-specific manner. Furthermore, *MDL1AS* may constitute a predictive biomarker and a molecular target for new therapies.

## Simple Summary

Tumoral heterogeneity is one of the main obstacles to fully understanding cancer biology. Long non-coding RNAs (lncRNAs) are gaining considerable interest as they are involved in many tumor-related processes. Therefore, we aimed to study the role of *mitochondrial D-loop 1 antisense lncRNA (MDL1AS)* in SH-SY5Y and NTERA2 cells, which represent cells with different grades of stemness and differentiation. We found that *MDL1AS* affects metabolism, differentiation, and response to radiation in a cell type-dependent manner. These data suggest that *MDL1AS* may hold potential as a predictive biomarker or even as a therapeutic target.

## 1. Introduction

Tumors are composed of different cell populations, and high levels of intratumoral heterogeneity negatively affect patient outcomes and complicate the prediction of treatment sensitivity [1,2,3]. Therefore, it is essential to understand the contribution of different tumor cell subpopulations to propose and validate new prognostic biomarkers for specific clinical contexts [4,5]. Among these populations, cancer stem cells (CSCs) have attracted particular interest due to their key role in tumor resistance and recurrence [6]. CSCs are characterized by a high degree of undifferentiation and plasticity, which enables them to dynamically transition between different cellular states, in contrast to more static populations that are more sensitive to drug treatments [7,8,9,10]. This plasticity critically influences the therapeutic outcome, particularly in response to radiotherapy [11].

A defining feature of CSCs is their ability to enter a quiescent state [12], which can alternate between invasive or proliferative phenotypes [13]. Another important process in treatment resistance is senescence, which is less common in CSCs, and in which cells do not recover proliferative capacity [14] but can promote proliferation and radioresistance of neighboring or distant tumor cells through the senescence-associated secretory phenotype (SASP) [15]. Both quiescence and senescence are associated with activation of the DNA damage response (DDR) or autophagic pathways [6], which contribute to radiotherapy resistance by promoting survival after radiation-induced DNA damage [16,17]. DNA repair pathways prevent DNA damage-induced cell death and apoptosis [6,18], often in a p53-dependent manner [5].

Cancer cell metabolism is another hallmark associated with CSCs and treatment response. Although many cancer cells undergo Warburg metabolism [19], CSCs often maintain oxidative phosphorylation (OXPHOS) as their main source of energy [20]. Metabolic preferences in CSCs are highly context-dependent and may confer selective advantages depending on tumor type; for instance, glioblastoma multiforme stem cells seem to rely primarily on OXPHOS [21,22], whereas hepatic CSCs preferentially utilize glycolysis [23]. Although differentiation-inducing therapies can increase treatment sensitivity, the impact of differentiation status on radiation resistance needs to be further elucidated [24]. Moreover, CSCs are associated with hypoxic tumor niches, which reduce reactive oxygen species (ROS)-mediated damage and favor glycolytic metabolism, further contributing to radioresistance.

In this context, long non-coding RNAs (lncRNAs) are attracting increasing interest due to their crucial roles in cancer biology. They could influence mitochondrial metabolism, cell proliferation, treatment resistance, and many other processes, and therefore hold promise as potential cancer biomarkers and therapeutic targets [25,26,27]. Furthermore, they may be relevant in understanding CSC plasticity and resistance acquisition [28,29].

Among the large number of lncRNAs, the *mitochondrial D-loop 1 antisense lncRNA* (*MDL1AS*) has attracted recent attention. This lncRNA is encoded by the mitochondrial circular DNA, more specifically by the antisense strand between positions 16,024 and 407, according to the human reference genome. *MDL1AS* is differentially expressed in several cancer models, compared to their normal counterparts [30]. Interestingly, this differential expression was context-specific, being upregulated in breast and larynx cancers but downregulated in tumors of the colon and rectum [31]. Furthermore, low levels of *MDL1AS* were a strong predictor of poor prognosis in colorectal cancer patients [31]. Another study showed the implications of *MDL1AS* in cell metabolism and its presence in both the nucleus and cytoplasm [32]. The fact that the sequence coding for *MDL1AS* bridges the mitochondrial replication origin, and that the official reference mitochondrial genome is split at that point, has precluded the analysis of the expression of this lncRNA with common transcriptomic informatics tools [30,31]. The few recent studies on this molecule suggest that it may provide a plethora of clinically relevant information in connection with important biological functions.

To avoid confusion, we must indicate that the lncRNAs *MDL1* and *MDL1AS*, which are coded in the mitochondrial genome [30,31], are not related to the protein MDL1, which is coded by the gene *CLEC5A*, located in human chromosome 7 [33].

Since the differentiation state of a cell is a major factor in therapy resistance, the aim of this study was to analyze the role of *MDL1AS* in two neural-like cancer cell models with different levels of stemness and differentiation. The first model, SH-SY5Y cells, was derived from a human neuroblastoma and exhibits a neuroblastic phenotype with stem-like features [34]. Despite their cancer origin, they are widely used as a neuronal model and have been used to study the effect of radiation therapy [35,36,37]. The other cell line, NTERA2, originated from a human testicular teratocarcinoma with germinal origin and consists of highly undifferentiated and pluripotent stem cells, commonly used to model neuroectodermal differentiation [38,39]. In addition, NTERA2 cells have also been used to study CSC biology and neural differentiation, and have been shown to be suitable for radiosensitivity testing [40,41,42]. Therefore, these complementary models allowed us to study the function of *MDL1AS* in tumor differentiation, metabolism, and radioresistance.

## 2. Materials and Methods

### 2.1. Cell Lines and Culture

The neuroblastoma cell line SH-SY5Y and the testicular teratocarcinoma cell line NTERA2 were obtained from the American Type Culture Collection (ATCC, Manassas, VA, USA), cultured in DMEM medium (Corning, New York, NY, USA) supplemented with 10% fetal bovine serum (FBS, HyClone, Logan, UT, USA), and maintained in a 37 °C environment, with an atmosphere containing 5% CO_2_ and 85% relative humidity.

### 2.2. Gene Downregulation

For physiology experiments, *MDL1AS* expression was downregulated with Dicer-substrate small interfering RNAs (DsiRNAs), as described [31]. DsiRNAs against *MDL1AS* were synthesized at a 10 nmol scale by Integrated DNA Technologies (IDT, Coralville, IA, USA) (Table 1). As a negative control, the AllStars Negative Control siRNA was used (1027280, Qiagen GmbH, Hilden, Germany) and was named siRNAnc for this study. Stock preparations were diluted in RNase-free water to a final concentration of 100 µM. For transfection, SH-SY5Y and NTERA2 cells were seeded in the corresponding plate at a cell density, depending on the experiment, and the following day they were transfected with Lipofectamine RNAiMAX (LPF, Thermo Fisher, Waltham, MA, USA) at a final concentration of 0.3% *v*/*v* in serum-free media and containing 10 nM DsiRNA. Gene silencing was confirmed after 48 h by qRT-PCR (see below) for the different experiments.

### 2.3. RNA Extraction and qRT-PCR

SH-SY5Y or NTERA2 cells were seeded in 6-well plates (3.5 × 10^5^ cells/well). The following day, cells were transfected with either DsiRNA2, DsiRNA3, or DsiRNA4. After 48 h, 500 µL of TRIzol (Invitrogen, Carlsbad, CA, USA) were added, and samples were frozen at −80 °C. RNA extraction was done using the NZY total RNA isolation kit (NZYtech, Lisbon, Portugal), according to the manufacturer’s instructions. cDNA synthesis was performed with NZY First-Strand cDNA Synthesis Kit (NZYtech), and quantitative PCRs were set up with NZYSupreme qPCR Green Master Mix (NZYtech) containing PCR primers (Table 2) at a final concentration of 0.3 µM. Results were generated in the QuantStudio 5 Real-Time PCR thermocycler (Applied Biosystems, Norwalk, CT, USA). Cycling conditions were 95 °C, 10 min, and 40 cycles of 95 °C, 15 sec, and 60 °C, 1 min. A final dissociation curve step from 60 to 90 °C was applied. The cDNA amount was established through interpolation into a standard curve specific for each gene. The data was analyzed with QuantStudio Design & Analysis Software v1.5.1 (Applied Biosystems). The housekeeping genes used for normalization were the nuclear gene, *glyceraldehyde-3-phosphate dehydrogenase* (*GAPDH*), and the mitochondrial gene, *NADH dehydrogenase 1* (*ND1*).

### 2.4. RNAseq

For RNAseq analysis, SH-SY5Y or NTERA2 cells were seeded in 6-well plates (1.5 × 10^5^ cells/well). After 24 h, cells were transfected with DsiRNA3 and DsiRNA4, respectively. Untransfected cells were used as a negative control. Two days after transfection, RNA was extracted, and qRT-PCR was performed to confirm *MDL1AS* expression reduction.

Then, RNA quality was evaluated through the Fragment Analyzer (AATI) and the Standard Sensitivity RNA Analysis Kit (15 nt) and quantified with Qubit 3.0 (Qubit^®^ RNA BR Assay Kit, Invitrogen, Carlsbad, CA, USA). Libraries were prepared with the TruSeq Stranded mRNA Kit (Illumina, San Diego, CA, USA), and the quality and quantity of these libraries were assessed again. The libraries were sequenced with NovaSeq6000 equipment (100-cycle paired-end format). Then, the quality of the reads was analyzed (FastQC v0.12.1), and reads were cleaned and trimmed (FastP v0.23.4). Then, reads were aligned to the *Homo sapiens* GRCh38 reference genome using HISAT2 (v2.2.1), and the quality of the alignments was again assessed (Qualimap v2.2.1). Finally, the quality reports were summarized with MultiQC (v1.21). Raw reads were counted (FeatureCounts v2.0.6). Principal Component Analysis (PCA) was performed to evaluate sample distribution (DESeq2 v1.42.0).

Differential expression analysis was performed using two methods: DESeq2 1.42.0 [43] and EdgeR v4.0.16 [44], both with SARTools v1.8.1 package [45] using R v4.3.3. Statistical significance was set with an alpha value = 0.05, and adjusted by the Benjamini–Hochberg FDR method. For downstream analysis, differentially expressed genes (DEG) were selected using Fold Change (FC) ≤ 0.685/≥ 1.46 (│log_2_FC│ ≥ 0.58). The terms and relevant pathways were evaluated using multiple enrichment analysis such as WikiPathway_2021_Human, GO_Biological_Process_2021, MGI_Mammalian_Phenotype_Level_4_2021, and ChEA_2022 from Enrichr [46], which are available at https://maayanlab.cloud/Enrichr/accessed on 16 October 2025. Other free software packages, such as gprofiler (https://biit.cs.ut.ee/gprofiler/ accessed on 16 October 2025) and DAVID (https://david.bioinformatics.nih.gov/accessed on 16 October 2025), were also used. Pathways were represented according to Z-score and adjusted *p*-values.

### 2.5. Western Blotting

SH-SY5Y or NTERA2 cells were seeded in p100 plates (5.5 × 10^5^ cells/plate). After 48 h, cells were transfected with DsiRNA3 or DsiRNA4, respectively. The LPF-only condition was used as a negative control. Four days after transfection, plates were washed twice with PBS and TIS medium [47] (RPMI medium (Corning) supplemented with 10 µg/mL human transferrin, 10 µg/mL insulin, and 0.05 nM sodium selenite, all from Sigma-Aldrich (St. Louis, MO, USA)), was added for 24 h. The following day, the media were collected and frozen at −20 °C, whereas the cells were lysed using Mammalian Protein Extraction Reagent (M-PER) (Thermo Scientific) supplemented with complete protease and phosphatase inhibitor cocktail (Roche Diagnostics, Basel, Switzerland).

Cell lysates were centrifuged, and the protein contents of the supernatant were quantified using the Bradford Protein Assay (BioRad, Hercules, CA, USA). TIS media were passed through Amicon Ultra 3 kDa filters (Merk Millipore, Burlington, MA, USA) to concentrate the secreted proteins and remove excess salts. Then, the remaining product was passed through Amicon Ultra 100 kDa filters (Merk Millipore) to remove large proteins. The flow-throughs of the second filtering contained proteins with molecular weights between 3 and 100 kDa, and these were freeze-dried (Telstar, Terrasa, Barcelona, Spain). Samples were resuspended in 26 µL distilled water prior to electrophoresis.

Nupage sample reducing buffer 10x (Invitrogen) and loading buffer 4× (NuPAGE LDS Sample Buffer, Invitrogen) were added to the lysates (30 µg total cellular protein) or to the concentrated conditioned media (26 µL), heated at 70 °C for 10 min, and separated by sodium dodecyl sulfate (SDS) polyacrylamide gel electrophoresis in 4–12% Bis-Tris gels (Invitrogen). The separated proteins were transferred to Polyvinylidene Fluoride (PVDF) membranes using the iBlot 2 Transfer Device (Invitrogen). Then, membranes were blocked using 5% (*w*/*v*) non-fat dry milk in Tris-buffered saline (TBS; 25 mM Tris, pH 7.5, 150 mM NaCl) for one hour and then incubated overnight with the primary antibody (Table 3) dissolved in TBS-T (TBS with 0.1% (*v*/*v*) Tween-20). On the second day, peroxidase-labeled secondary antibodies were applied, and peroxidase activity was detected with the NZY Advanced ECL Western Blotting Detection Reagent (MB40201, Lisbon, Portugal) and captured in a ChemiDoc MP Imaging System (BioRad). Quantification of immunoreactive bands was performed using ImageJv1.53t, and normalization was done with GAPDH expression for the cellular proteins and with total protein quantification for secreted proteins.

### 2.6. Viability Assay

To analyze proliferation and viability, the MTS technique was used, as described [48]. SH-SY5Y or NTERA2 cells were seeded in 96-well plates (Falcon) (3000 cells/well). The following day, cells were transfected with DsiRNA3 or DsiRNA4, respectively, and the LPF-only condition was used as a negative control. Twenty µL of CellTiter 96^®^ Aqueous One Solution Cell Proliferation Assay (Promega, Madison, WI, USA) were added per well at 0, 24, 48, 72, 96, and 120 h following *MDL1AS* silencing, incubated for 4 h, and the absorbance at 490 nm was recorded in a POLARstar Omegav1.20 (BMG Labtech, Ortengerb, Germany) plate reader. Each condition was normalized against its value at time 0.

### 2.7. Irradiation Assays

For irradiation assays, SH-SY5Y (12,500 cells/well) and NTERA2 (7000 cells/well) cells were seeded in 96-well plates. The following day, they were transfected as before and, two days later, they were subjected to ionizing radiation in the CellRad X-ray irradiator (Faxitron, Tucson, AZ, USA). Each of the transfection conditions was subjected to 0, 2.5, 5, and 10 Gy of radiation using the auto-dose control mode, with constant 138 kV and 5.95 mA for the time required to reach the specified dose. After that, cells were kept in their incubator until the next day, when the MTS assay was done to evaluate cell viability.

For RNA expression analyses, cells were seeded in p100 plates, and once the cells had reached the proper density, they were irradiated with 5 Gy, maintaining the rest of the parameters constant. Then RNA was extracted 2 and 48 h after irradiation to evaluate early and late-transcriptional changes on *MDL1AS* levels by qRT-PCR, as above.

### 2.8. Mitochondrial Metabolism

Mitochondrial metabolism was assessed using the Seahorse XFe24 Analyzer (Agilent, Santa Clara, CA, USA), specifically the ATP Rate Assay (Agilent), following the manufacturer’s instructions. Briefly, SH-SY5Y (30,000 cells/well) or NTERA2 (20,000 cells/well) cells were seeded in Seahorse’s 24-well plates. The following day, cells were transfected with the corresponding DsiRNA. The LPF-only condition was used as a negative control. On the third day, the assay cartridge was hydrated with Seahorse XF Calibrant Solution (Agilent) and incubated overnight at 37 °C without CO_2_. On the day of the experiment, cells were washed with Seahorse XF DMEM medium, pH 7.4, supplemented with 10 mM glucose, 1 mM pyruvate, and 2 mM L-glutamine (Agilent). A total volume of 500 µL of this medium was then added to each well for 1 h incubation at 37 °C without CO_2_. The following inhibitors were used for the assay: oligomycin (1.5 µM) and a mixture of Rotenone/antimycin A (0.5 µM each) (Agilent). Data were acquired and analyzed using Seahorse Wave Software v2.6.3. Data normalization was performed by calculating total protein concentration per well by BCA assay (Thermo Fisher Scientific, Waltham, MA, USA) at the end of the experiment, according to the manufacturer’s instructions.

### 2.9. Retinoic Acid-Induced Cell Differentiation

SH-SY5Y and NTERA2 cells were seeded in 6-well plates (6–40 × 10^4^ cells/well) in the absence or presence of 10 µM retinoic acid (RA, Sigma-Aldrich) in medium containing 2% FBS and 1% Pen/Strep (Gibco). The following day, cells were transfected with DsiRNA3 or DsiRNA4, respectively. Three, 6, and 8 days after transfection, the medium was renewed with or without RA. Cells were passaged when needed to maintain an appropriate density. On day 10 after transfection, photographs were taken with a DMI4000B inverted microscope (Leica Microsystems, Wetzlar, Germany). The number of neurites per cell was manually counted and calculated for each condition with the help of the ImageJ free software.

### 2.10. Statistical Analysis

After ensuring that datasets followed a normal distribution, the *t*-test was used for comparisons between two groups, and one- or two-way ANOVA followed by Tukey’s correction for more than two groups. R (v4.5.1) and GraphPad (8.4.2) were used to do statistical analyses and data representations. *p* < 0.05 was used as the significance threshold. Graphics are represented with mean and standard deviation (SD), and all tests were two-tailed unless otherwise specified.

## 3. Results

### 3.1. MDL1AS Expression Is Downregulated by Different DsiRNAs

We started the study by selecting the DsiRNA candidate (Table 1) that downregulated *MDL1AS* expression more consistently in either NTERA2 or SH-SY5Y cells. We tested gene expression at different periods and found that a significant downregulation was obtained two days after transfection, with no noticeable differences when waiting for a third or fourth day. In a preliminary test, all DsiRNAs induced reductions in *MDL1AS* levels in both cell lines, being more effective in SH-SY5Y cells (Table 4). These data were obtained using the second pair of primers for *MDL1AS* (Table 2, primers 2), normalized against *ND1*.

In both cell lines, a commercial negative control (siRNAnc) was compared against the medium-only control and lipofectamine alone (LPF), and no significant differences in *MDL1AS* levels were observed (Figure S1).

### 3.2. MDL1AS Downregulation Affects the DDR, Senescence, Autophagy, and Apoptosis Pathways on the NTERA2 Cell Line

**Figure 1:**
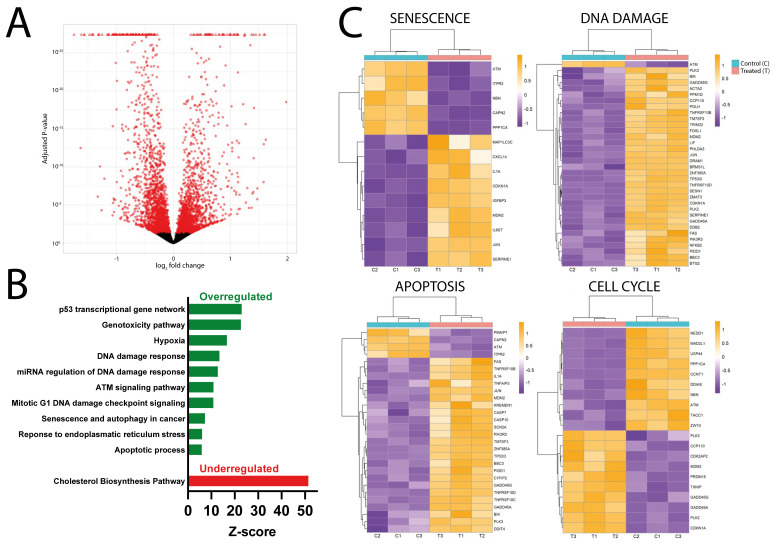
Transcriptomic analysis results of the reduction in *MDL1AS* levels in NTERA2 cells. (**A**) Volcano plot showing DEGs of the DsiRNA group versus the medium-only control. Each dot represents a gene that is either differentially expressed (red) or not (black) (n = 3). The *X*-axis represents the log_2_FC, and the *Y*-axis represents the adjusted *p*-value. (**B**) Bar graph of the main enriched pathways between the DsiRNA and control groups. The most highly modulated pathways are shown as overexpressed (green) or underexpressed (red) in the DsiRNA group. The X-axis represents the Z-score obtained from the Enrichr analysis. (**C**) Selected heatmaps showing the main DEGs in DsiRNA and control groups within specific altered pathways: senescence, DDR, apoptosis, and cell cycle (n = 3). Every row corresponds to a DEG, and the color of each square represents its log_2_FC (orange for positive values and purple for negative values). Control columns are indicated in blue, whereas the DsiRNA group is shown in brick red.

DsiRNA4 was chosen as the most reliable regulator in the case of NTERA2 cells. To investigate the potential cellular pathways that may be modulated by the downregulation of *MDL1AS* expression, treated and untreated triplicate samples were subjected to RNAseq analysis. We first analyzed the 4525 DEGs between both conditions (DESeq2, 2173 upregulated genes and 2352 downregulated genes), which are represented in red in a volcano plot (Figure 1A). After analyzing both cell line results (SH-SY5Y results are shown below), │0.58│ log_2_FC was selected as the optimum expression threshold.

Then, we used the Enrichr analysis to evaluate which pathways were affected by the alteration of *MDL1AS* levels. The main upregulated pathways were the DDR and, in general, pathways related to responses to stimuli (Figure 1B). Specifically, these pathways included the p53-mediated transcriptional network, DNA and miRNA regulation of DDR, hypoxia, genotoxicity pathways, or the ATM signaling system. Other affected pathways were senescence, autophagy, and apoptosis, which, to a certain extent, are also associated with the DDR (Figure 1B). Additional pathways overregulated in DsiRNA conditions were the ForkHead Box O (FOXO) signaling and the Nuclear Factor kappa B (NF-κB) pathway. The main underregulated pathway was the cholesterol biosynthesis pathway, followed by the mevalonate or fatty acid biosynthesis.

Some of the DEGs associated with these upregulated pathways, namely senescence, DDR, apoptosis, and cell cycle, are further represented by heatmaps (Figure 1C).

### 3.3. MDL1AS Downregulation Affects the DDR and Apoptosis Pathways on the SH-SY5Y Cell Line

**Figure 2:**
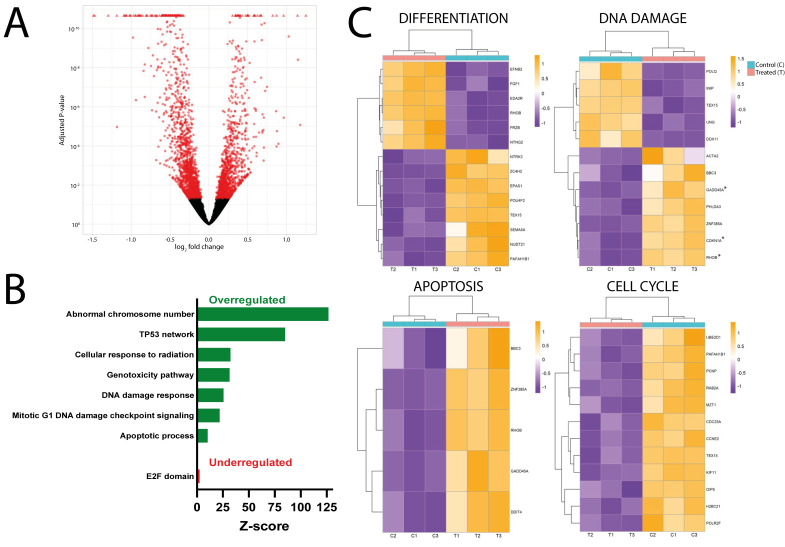
Transcriptomic analysis results of the reduction in *MDL1AS* levels in SH-SY5Y cells. (**A**) Volcano plot comparing the DsiRNA3-treated versus the control group. Each dot represents a gene that is either differentially expressed (red) or not (black) (n = 3). The *X*-axis represents the log_2_FC, and the *Y*-axis represents the adjusted *p*-value. (**B**) Bar graph of the main enriched pathways between the DsiRNA and control groups. The most highly modulated pathways are shown as overexpressed (green) or underexpressed (red) in the DsiRNA group. The X-axis represents the Z-score obtained from the Enrichr analysis. (**C**) Selected heatmaps showing the main DEGs in the DsiRNA and control groups within specific altered pathways: differentiation, DDR, apoptosis, and cell cycle (n = 3). Every row corresponds to a DEG, and the color of each square represents its log_2_FC (orange for positive values and purple for negative values). Control columns are indicated in blue, whereas the DsiRNA group is shown with brick red. Asterisks identify genes involved in radiation response.

Regarding the SH-SY5Y, DsiRNA3 was selected as the optimal expression repressor. The representation of the DEGs (red) between the DsiRNA-treated and the control samples in a volcano plot showed 3013 modified genes (DESeq2, 1354 upregulated genes and 1659 downregulated genes) (Figure 2A). The representation of the log_2_FC has a narrower aspect than that of the NTERA2 cells (Figure 1A), showing that the variation in the number of DEGs is smaller in the former.

Next, we represented the main upregulated and downregulated pathways in the DsiRNA group, as calculated by Enrichr analysis. In this case, genes associated with abnormal chromosome number are the most represented group. Furthermore, we found many genes related to DNA damage and noxious stimuli, including the p53 network, genotoxicity pathway, DDR, mitotic G1 DNA damage checkpoint stimuli, and the apoptotic process. Another important pathway that was upregulated in SH-SY5Y cells was the cellular response to radiation (Figure 2B). With a lower representation, we also found the FOXO-mediated transcription of genes. A small number of genes associated with the E2F pathways or protein complex involved in cell adhesion were downregulated (Figure 2B).

Finally, some of the DEGs associated with these dysregulated pathways, namely differentiation, DDR, apoptosis, and cell cycle, are represented by heatmaps (Figure 2C). Interestingly, some of the genes on the DDR panel are also involved in the response to radiation exposure (asterisks next to their names in Figure 2C).

### 3.4. MDL1AS Downregulation Induces Radiation Resistance in NTERA2 but No Clear Effect Is Seen in SH-SY5Y Cells

**Figure 3:**
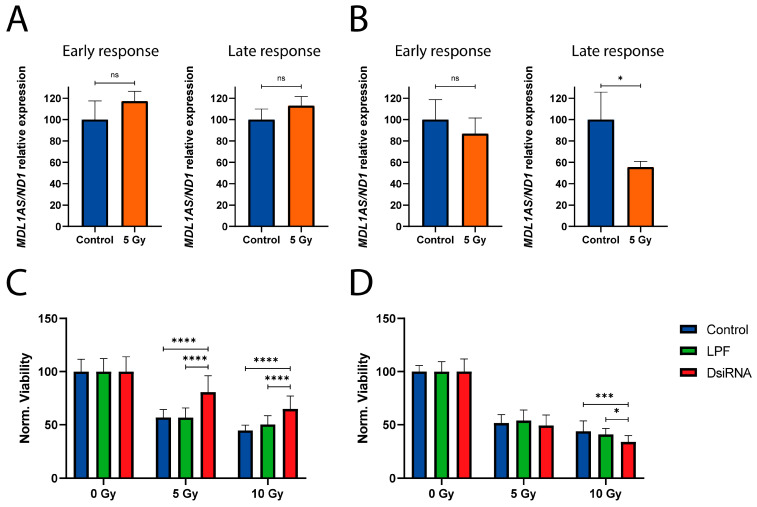
Effects of radiation on *MDL1AS* expression and cell survival. (**A**,**B**) *MDL1AS* expression of NTERA2 (**A**) and SH-SY5Y cells (**B**) after 2 h (left) or 48 h (right) of exposure to 5 Gy of radiation (orange), compared to unirradiated medium-only controls (blue) (n = 3). *MDLA1AS* expression was quantified using the second *MDL1AS* pair of primers and normalized to *ND1*. Then, all values were expressed relative to the non-irradiated condition (Blue). Statistical analysis was performed using Unpaired *t*-test. (**C**,**D**) Cell viability of the different groups: medium-only control (blue), LPF control (green), and DsiRNA-treated samples (red) of NTERA2 (**C**) and SH-SY5Y (**D**) cells. Samples were collected 24 h after exposure to 0, 5, and 10 Gy of radiation. Cell viability was estimated by the MTS protocol (3 IEs, n = 28). Each of the irradiated conditions was normalized to the non-irradiated group with the same pretreatment. Statistical analysis was performed using two-way ANOVA followed by Tukey’s multiple comparisons test. ns: non-significant; *: *p* < 0.05; ***: *p* < 0.001; ****: *p* < 0.0001.

After observing that downregulation of *MDL1AS* increases the expression of genes associated with the DDR at different levels (p53, cell cycle checkpoints, senescence, apoptosis, or response to hypoxia), we evaluated this phenomenon at the physiological level. First, we exposed cells to radiation to see whether *MDL1AS* expression is altered. Two hours (early transcriptional response) or forty-eight hours (late transcriptional response) after exposure to 5 Gy, we did not observe any significant change in *MDL1AS* expression in NTERA2 cells (Figure 3A). In irradiated SH-SY5Y cells, we did not observe changes in *MDL1AS* levels after 2 h, but after 48 h, we did find a significant decrease in expression in the irradiated SH-SY5Y cells of 44.4 ± 5.3% (*p* = 0.0430) (Figure 3B).

Our second question was whether silencing *MDL1AS* expression could induce changes in the viability of cells exposed to radiation. To check this, medium-only control cells, cells treated with LPF, and DsiRNA-treated cells were exposed to different doses of radiation, and viability was observed after 24 h. In NTERA2 cells, we observed that *MDL1AS* reduction with DsiRNA4 induced resistance, e.g., lower viability reduction in silenced cells after radiation with 5 Gy (C: 43.2 ± 7.5 vs. LPF: 43.3 ± 9.2 vs. DsiRNA4: 19.4 ± 15.5%; *p* < 0.0001) and 10 Gy (C: 55.3 ± 5.0 vs. LPF: 49.7 ± 8.2 vs. DsiRNA4: 35.2 ± 12.3%; *p* < 0.0001) (Figure 3C). However, we did not see changes in SH-SY5Y cells (treated with DsiRNA3) when exposed to 5 Gy of radiation, but we observed the opposite effect with 10 Gy (C: 56.2 ± 9.7 vs. LPF: 59.1 ± 5.5 vs. DsiRNA3: 66.0 ± 5.9%; *p* = 0.0166), as cells were more sensitive to radiation and showed less viability with DsiRNA3 (Figure 3D).

To confirm the results, the experiment was repeated with the remaining DsiRNAs in both cell lines. Results obtained from NTERA2 cells show a significantly increased viability in DsiRNA-treated cells, same as described with DsiRNA4. The viability reduction against the non-irradiated cells in the different conditions with 5 Gy (C: 37.9 ± 4.4 vs. LPF: 40.3 ± 8.1 vs. DsiRNA2: 30.7 ± 14.7; *p* < 0.0001; vs. DsiRNA3: 24.9 ± 9.9%; *p* < 0.0001) and with 10 Gy (C: 38.6 ± 7.3 vs. LPF: 37.6 ± 8.0 vs. DsiRNA2: 22.3 ± 6.9; *p* < 0.0001; vs. DsiRNA3: 23.2 ± 9.3%; *p* < 0.0001) is represented in Figure S2A.

However, no significant changes in viability were observed in SH-SY5Y cells (Figure S2B), confirming a different behavior between both cell lines but not reproducing the results obtained with DsiRNA3.

### 3.5. MDL1AS Downregulation Reduces PAI-1 Secretion in NTERA2 Cells and Increases Lamin B1 Protein Expression in SH-SY5Y Cells

Among the DEGs found in the transcriptomic analysis, we focused on two genes/proteins related to radiosensitivity or senescence in the literature. On the one hand, *SERPINE1*, which codes for PAI-1 (plasminogen activator inhibitor 1), is known to increase as a response to radiation-induced DNA double-strand breaks and facilitates DNA repair in an ATM/ATR-dependent manner [49]. In addition, its overexpression correlates with poor outcomes and radioresistance [50]. Furthermore, the reduction in *SERPINE1* expression reduces aggressiveness and radioresistance in triple-negative breast cancer [49]. Apart from its role in DDR and senescence, PAI-1 is also part of the SASP [51,52,53,54]. On the other hand, Lamin B1 (coded by *LMNB1*) has implications in radioresistance as it prevents Radiation sensitive protein 51 (RAD51) proteosome degradation, and its downregulation sensitizes U2OS cells to radiation [55].

**Figure 4:**
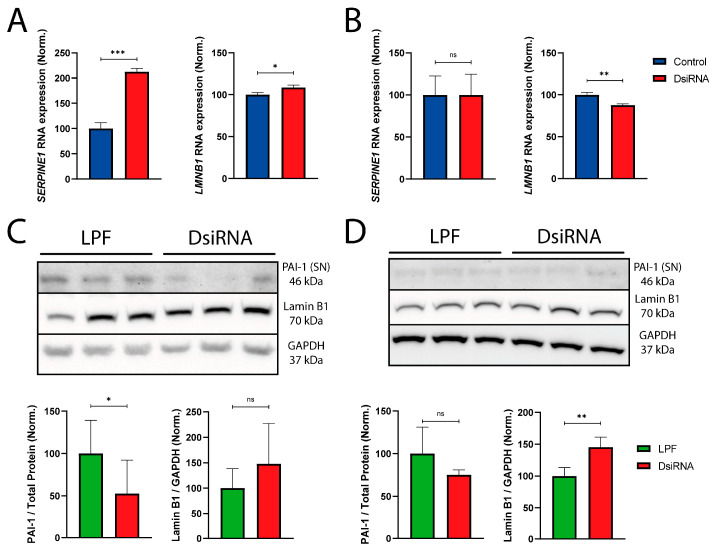
RNA and protein expression of *SERPINE1* and *LMNB1* in both cell lines. (**A,B**) *SERPINE1* and *LMNB1* RNA normalized expression on DsiRNA conditions (red) compared to untreated controls (blue) in NTERA2 (**A**) and SH-SY5Y cells (**B**). Data were obtained from the RNA seq analysis, and normalized counts per sample were used for the analysis (n = 3). Values from the DsiRNA condition were expressed relative to the control (blue). Statistical analysis was obtained by FDR methods. (**C**,**D**) Representative image of Western blots comparing cells treated with LPF as a control (left) to those treated with DsiRNA (right), in triplicate in NTERA2 (**C**) and SH-SY5Y cells (**D**). The PAI-1 band was obtained from freeze-dried serum-free conditioned medium, whereas the Lamin B1 and GAPDH bands were from cell lysates. Approximate band sizes are 46, 70, and 37 kDa, respectively. PAI-1 (left) and Lamin B1 (right) protein expression on DsiRNA condition (red) against LPF control (green) in NTERA2 (**C**) (PAI-1, 3 IEs, n = 7; Lamin B1, 2 IEs, n = 4) and SH-SY5Y cells (**D**) (PAI-1, 1 IE, n = 3; Lamin B1, 2 IEs, n = 4). Each protein expression value was normalized by GAPDH expression, and all values were expressed relative to the LPF control (green). Statistical analysis was performed using Unpaired *t*-test. ns: non-significant; *: *p* < 0.05; **: *p* < 0.01; ***: *p* < 0.001.

We first investigated the RNA expression levels of these two genes by RNA-Seq. In NTERA2 cells, *SERPINE1* showed a significant overexpression in the DsiRNA group compared to the control (*p* < 0.001), and *LMNB1* was also slightly overexpressed (*p* = 0.0180) (Figure 4A). In SH-SY5Y cells, no changes were seen for *SERPINE1* levels, whereas *LMNB1* was downregulated (*p* = 0.0028) (Figure 4B).

Surprisingly, when we looked at the protein expression levels, the results were rather different. PAI-1 is secreted extracellularly and was quantified in serum-free conditioned medium. Under these conditions, the levels of PAI-1 were significantly reduced by 47.1 ± 39.3% (*p* = 0.0446) in the DsiRNA-treated NTERA2 cells (Figure 4C) while the levels of secreted protein in SH-SY5Y cells showed no significant differences (Figure 4D). In the case of Lamin B1, no significant changes were found in NTERA2 cells (Figure 4C), but there was a significant increase of 45.5 ± 15.7% (*p* = 0.0045) in DsiRNA-treated SH-SY5Y cells (Figure 4D). Full blot images are available in Figure S3.

### 3.6. MDL1AS Downregulation Decreases Tumor Cell Growth in NTERA2 and SH-SY5Y and Increases Glycolytic Status in NTERA2 Cells

**Figure 5:**
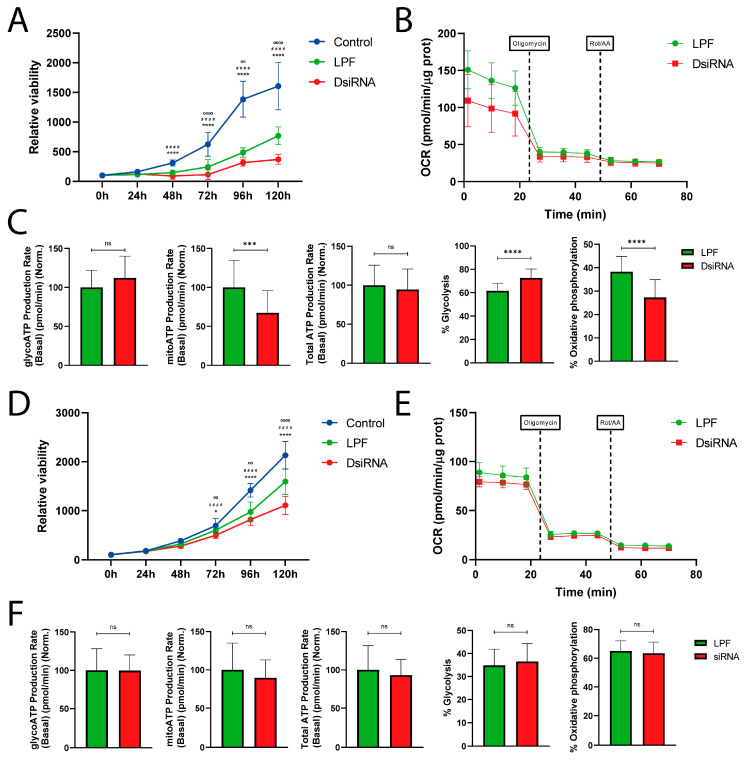
Effects of *MDL1AS* downregulation on NTERA2 (**A**–**C**) and SH-SY5Y (**D**–**F**) cell growth and metabolism. (**A**,**D**) Cell viability of the different groups: untreated control (blue), LPF control (green), and DsiRNA4-treated (red) in NTERA2 (**A**) and DsiRNA3-treated (red) in SH-SY5Y cells (**B**) at different times after gene silencing. Cell number was estimated with the MTS protocol (NTERA2 5 IEs, n = 40/SH-SY5Y 3 IEs, n = 24). Each of the times was normalized to the 0 h condition with the same pretreatment. Statistical analysis was performed using two-way ANOVA followed by Tukey’s multiple comparisons test. * symbol represents the statistical significance for the control vs. LPF comparison at each time, #### symbol represents the control vs. DsiRNA4 or DsiRNA3 comparison, and ºº and ºººº symbols are used for the LPF vs. DsiRNA4 or DsiRNA3 comparison. (**B**,**E**) Representative graph showing the OCR in an ATP Rate assay of LPF- (green) and DsiRNA4-treated (red) NTERA2 cells (**B**) or DsiRNA3-treated (red) SH-SY5Y cells (**E**). Oligomycin (at 24 min) and Rotenone/Antimycin A (at 48 min) injections are indicated. (**C**,**F**) Different metabolic parameters derived from the interpretation of the OCR and ECAR of the ATP Rate assay for NTERA2 (**C**) and SH-SY5Y cells (**F**). Statistical analysis was performed using Unpaired *t*-test (3 IEs, n = 25). ns: non-significant; *: *p* < 0.05; ºº: *p* < 0.01; ***: *p* < 0.001; #### or ºººº or ****: *p* < 0.0001.

The effect of silencing *MDL1AS* expression on the proliferation of NTERA2 and SH-SY5Y cells with DsiRNA4 and DsiRNA3, respectively, was also analyzed through MTS assays at different time points. First, we found that LPF by itself was able to significantly reduce cell growth when compared to untreated control cells (*p* < 0.0001). Nevertheless, DsiRNA-treated cells showed an even lower proliferation than the LPF control cells (*p* < 0.0001) (Figure 5A,D), indicating a significant antiproliferative effect of the lncRNA.

The metabolism of the NTERA2 cells following *MDL1AS* silencing was also studied by performing an ATP rate assay. The OCR of both conditions (basal, post-oligomycin, and post-Rot/AA) is shown (Figure 5B). With data from the OCR and the extracellular acidification rate (ECAR), different parameters related to ATP production were calculated. No significant differences were found between the experimental treatments in the case of glycoATP production rate (Figure 5C) or total ATP production rate (Figure 5C). On the other hand, the DsiRNA4-treated cells had a lower mitoATP production rate (*p* < 0.001) and percentage of OXPHOS (*p* < 0.0001) and a concomitantly higher glycolytic metabolism (*p* < 0.0001) (Figure 5C).

To further evaluate this effect, we repeated the ATP rate experiment with NTERA2 cells treated with DsiRNA2 and DsiRNA3 (Figure S4). No significant differences were found in the total or mitoATP production rates between the DsiRNA or LPF-only treated samples (Figure S4C,D). Neither were they found on glycoATP production rate on DsiRNA2-treated cells (Figure S4C). However, significant increases in glycoATP production rate were detected in DsiRNA3-treated samples (*p* < 0.05) (Figure S4D). Also, an increased percentage of glycolysis and concomitant lower percentage of OXPHOS were found in the DsiRNA2 and DsiRNA3-treated samples (*p* < 0.05) (Figure S4C,D).

Regarding mitochondrial metabolism of SH-SY5Y cells, no significant changes were elicited by the silencing of the *MDL1AS* gene with any of the DsiRNAs (Figure 5E,F and Figure S5A–D).

### 3.7. MDL1AS Downregulation Favors RA-Induced Neuronal Differentiation in SH-SY5Y Cells but Not in NTERA2 Cells

**Figure 6:**
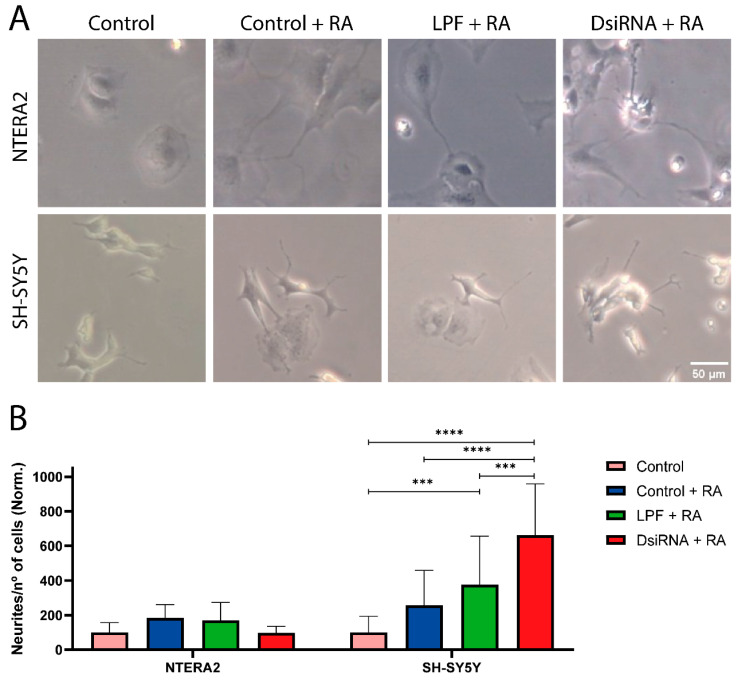
Effects of *MDL1AS* downregulation on RA-induced neurite production. (**A**) Representative images of NTERA2 and SH-SY5Y cells treated with the indicated conditions to study differentiation. Cells were treated with 10 µM RA for 10 days. Photographs were made with an N PLAN 10x dry objective and 0.25 numerical aperture. Scale bar = 50 µm. (**B**) Number of neurites per cell under different conditions. Each condition was normalized to the medium-only control. Statistical analysis was performed using two-way ANOVA followed by Tukey’s multiple comparisons test (NTERA2, 2 IEs, n = 21; SH-SY5Y, 1 IE, n = 6). ***: *p* < 0.001; ****: *p* < 0.0001.

Since both cell lines can differentiate into neurons with the proper cues [56,57,58] and some RNAseq results suggest that silencing *MDL1AS* may affect differentiation, we wanted to analyze whether *MDL1AS* may interfere with this process by treating the cells with RA and counting the number of neurites per cell (Figure 6A). Representative images of the experiment show the appearance of neuritic prolongations in response to RA-treatment in NTERA2 (upper photos) and SH-SY5Y (lower photos), whereas control cells have lower levels of neurite formation.

Quantification of the number of neurites under different conditions (Figure 6B) showed no significant changes among NTERA cells. However, the results on SH-SY5Y indicate a significant increase in the number of neurites per cell on the LPF control over the untreated condition (*p* < 0.001). Furthermore, DsiRNA-treated cells responded better (*p* < 0.001) to the presence of RA than the LPF control, thus indicating that a more potent neural-like differentiation was activated by the lower levels of the lncRNA (Figure 6B).

## 4. Discussion

LncRNAs have been associated with cancer hallmarks, but many of them remain poorly understood, especially their regulation. Some of the cancer pathways in which lncRNAs participate are cell stemness, metabolic disorders, and DDR, among many others [59].

The recently discovered lncRNA, *MDL1AS*, is associated with metabolism and mitochondrial functions [32]. *MDL1AS* is also relevant as a cancer biomarker, although following a complex pattern. For instance, this lncRNA is overexpressed in tumor cells of the breast and the larynx when compared to normal cells of the same organ. Conversely, colorectal cancer cells have a lower expression of *MDL1AS* than normal colon cells. Consequently, patients diagnosed with locally advanced colorectal cancer who present high levels of *MDL1AS* have significantly better survival rates than patients with lower levels [31]. The same study demonstrated that *MDL1AS* downregulation decreased ATP production and oxygen consumption in both breast and colorectal cancer, although its influence on tumor cell proliferation was highly cell-type-dependent [31].

Based on the previous data, we hypothesized that *MDL1AS* may be involved, not only in cancer cell metabolism, but also in cell plasticity and stemness, and it may even play a role in therapy resistance. Therefore, the aim of this study was to discover new functions of *MDL1AS* in the context of two cell lines that present different levels of plasticity and differentiation. Hence, two diversely differentiated cell lines were selected: the NTERA2 cells with a high pluripotent potential [60] and the SH-SY5Y cells, a more differentiated, neurotypical cell line [61].

To confirm the adequate selection of the cell models, we checked the mitochondrial metabolism of these cells under basal conditions. We found that NTERA2 cells preferably use glycolysis to obtain ATP (60% glycolysis vs. 40% OXPHOS), whereas SH-SY5Y cells rely more on OXPHOS (35% glycolysis vs. 65% OXPHOS). Several studies have shown how the differentiation process is accompanied by a metabolic switch to OXPHOS [62,63], which supports the assumption that SH-SY5Y are more differentiated than NTERA2 cells. In the same line of thought, we can also point to the higher basal proliferation rate of NTERA2 over the SH-SY5Y cells, probably relying on a larger number of CSCs with highly proliferative capacities in the former [64].

Despite the different cellular models, we found many pathways that were commonly upregulated in both cell lines following *MDL1AS* reduction. These included the DDR, p53-ATM response, and apoptotic induction. In addition, the *Cyclin-dependent kinase inhibitor 1A (CDKN1A)* tumor suppressor is another commonly upregulated transcript. These results might suggest that *MDL1AS* is a cellular response switch repressor, and upon *MDL1AS* knockdown, the cells may trigger the defense pathways. These damage response pathways seem wider in NTERA2 cells, according to the RNAseq, where the number of DEGs following *MDL1AS* knockdown is higher, and these include pathways such as senescence, autophagy, hypoxia signaling, or response to endoplasmic reticulum stress. However, further research is needed to evaluate the correlation of the specific pathways with the general phenotypes found in this study.

To test this hypothesis, we subjected the cells to ionizing radiation. First, we found that SH-SY5Y cells experienced a significant reduction in *MDL1AS* expression 48 h after irradiation, whereas NTERA2 cells did not modify their *MDL1AS* levels under the same conditions. This result suggests that SH-SY5Y should induce their damage response pathways and become more resistant to radiation. However, the observed phenotypes discussed in the next paragraphs show the reverse pattern.

Evaluating cell survival after irradiation, we observed that NTERA2 cells became more resistant following *MDL1AS* downregulation with the three different DsiRNAs. In contrast, *MDL1AS* downregulation did not induce consistent changes in SH-SY5Y cells. In this case, we found radiosensitization with DsiRNA3, but this phenotype could not be reproduced with DsiRNA2 and DsiRNA4. Taken together, these data suggest a context-dependent action of *MDL1AS* downregulation, which is also supported by metabolic data, as shown below.

To evaluate the senescent response, as part of the hypothetic DDR behind this effect, we wanted to evaluate the radioresistance-related genes *SERPINE1* and *LMNB1* and their proteins [55,65,66,67]. In NTERA2 cells, the levels of *LMNB1,* or its protein Lamin B1, were not changed after *MDL1AS* reduction. In contrast, *SERPINE1* RNA expression was significantly increased under these conditions, but the protein quantification of its secreted protein, PAI-1, was significantly downregulated. These apparently contradictory observations may be due to complex posttranslational regulatory mechanisms that need to be eventually addressed.

In general, PAI-1 is secreted to the interstitial fluid as part of the SASP under cancer conditions, and it binds in a paracrine manner to specific receptors in the membrane of neighboring cells. These receptors include LRP1, which induces phosphorylation of AKT and ERK in the cytoplasm, leading to an increase in radioresistance [50,66]. Thus, in theory, the decrease in PAI-1 we observed in NTERA2 cells with lower levels of *MDL1AS* should have resulted in a radioresistance reduction, but we found the contrary results, indicating that PAI-1 does not seem mechanistically linked to the observed radioresistance.

SH-SY5Y cells showed a different behavior. *SERPINE1* or PAI-1 levels were unchanged by the downregulation of *MDL1AS*. In contrast, *LMNB1* RNA levels were reduced, whereas Lamin B1 protein expression was significantly upregulated following *MDL1AS* knockdown. This disconnection between the RNA and protein expression is similar to the mechanism described above for NTERA2 cells and PAI-1 and may also be related to posttranslational processes that need to be better understood to identify the exact mechanism underlying this observation. Lamin B1 is an intermediate filament that epigenetically modifies other genes’ expression. The loss of Lamin B1 expression is associated with cellular senescence [67]. The increase in Lamin B1 we saw in SH-SY5Y cells following *MDL1AS* downregulation should correlate with more proliferation and radiosensitization, but again, the levels of Lamin B1 do not fully correlate with our observed results, suggesting that neither PAI-1 nor Lamin B1 is mechanistically linked to the *MDL1AS* observed effects.

Since these cellular responses often depend on the metabolic state of the cell, and previous studies have shown that *MDL1AS* reduction induced metabolic changes [31], we further studied the metabolic response following *MDL1AS* reduction. It is known that CSC-specific characteristics are associated with metabolism [20]. In our study, NTERA2 cells were shown to rely more on glycolysis in their basal state than SH-SY5Y cells, and *MDL1AS* reduction induced a further increase in glycolytic ATP dependency with all the DsiRNAs tested. This glycolytic metabolism is highly associated with radioresistance, as widely described in the literature [68,69,70]. This association might occur because glycolysis favors DDR and redox regulation [71], but also because mitochondrial OXPHOS generates more ROS, making cells more sensitive to the damage caused by irradiation [72]. The protection against radiation damage granted by glycolysis to NTERA2 cells may be more potent than the induction of a certain radiosensitivity generated by the reduction in PAI-1 levels, as discussed above.

In contrast, the mitochondrial metabolism of SH-SY5Y cells remained unaltered after *MDL1AS* reduction induced by the three DsiRNAs. This observation strengthens the idea of cell line-dependent effects of *MDL1AS*. Furthermore, the lack of metabolic changes in SH-SY5Y following *MDL1AS* downregulation correlates with the general lack of changes in radiosensitivity for this cell line.

Given the tight relationship between metabolism and stemness, we wanted to analyze whether *MDL1AS* levels may affect differentiation or CSC markers expression. First, we investigated whether *MDL1AS* influences differentiation-related programs in the enriched pathways of RNAseq, including relevant CSC genes (Table S1) and neuron-related markers (Table S2). After discarding several of these markers because they were not expressed in our cells, we analyzed the levels of the remaining markers in our samples (Table S3).

Interestingly, when looking at untreated cells, all 10 CSC-associated genes are highly expressed in NTERA2 cells when compared to SH-SY5Y cells (Table S3, upper part). On the other hand, all six neuronal differentiation-linked genes are more expressed in SH-SY5Y cells when compared to NTERA2 cells (Table S3, lower part). These results confirm that NTERA2 cells have a higher expression of CSC markers than SH-SY5Y (which do not even express some of them) while SH-SY5Y cells show higher levels of neuronal markers, thus supporting the selection of these cells as experimental models of divergent differentiation.

Furthermore, *MDL1AS* downregulation does not seem to be affecting much the expression of CSC markers in any of the cell lines, but it is significantly increasing the expression of some neuritic markers (DCX, MAP1B, MAP2, and SYP) in the SH-SY5Y cell line (Table S3). This observation suggests a correlation between *MDL1AS* expression and the inhibition of neuronal fate.

Some of the CSC markers present in NTERA2 cells have functions that correlate with the observed phenotype. For example, high levels of *CD133* are related to enhanced DNA repair in gliomas or lung tumors and are associated with radioresistant non-small cell lung cancer [73]. In addition, *CD44* has been associated with recurrence and metastasis [73] and *ALDH1A1* has also been associated with radioresistance [74] and a more glycolytic metabolism [75]. These data correlate with our results, where we observe an increased radioresistance in NTERA2 cells subjected to *MDL1AS* downregulation, probably based on their increased stemness and glycolytic metabolism in comparison to SH-SY5Y cells.

To validate the hypothesis that *MDL1AS* expression modulates differentiation, we evaluated the impact of *MDL1AS* reduction on RA-induced neurite formation. Although it is possible to differentiate NTERA2 cells with RA [57], we found that *MDL1AS* downregulation did not change this situation. A possible explanation is that the higher levels of stemness found in these cells may keep them unaffected, or they may need larger exposure times. However, *MDL1AS* reduction significantly increased RA-induced neurite formation in SH-SY5Y cells, a clear hallmark of differentiation in these cells [56].

**Figure 7:**
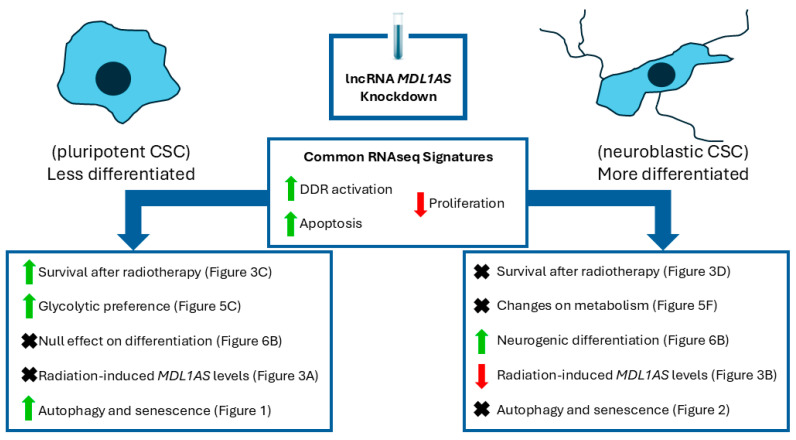
Overview of the results. Cartoon showing the common features observed in both cell lines (NTERA2 and SH-SY5Y) after *MDL1AS* knockdown. On the left-hand side, the effects of the downregulation on the NTERA2 cells (undifferentiated model) are shown. On the right-hand side, the changes in the SH-SY5Y (more differentiated model) are represented. Green arrows indicate increases whereas red arrows show decreases.

The higher neurogenic activity of SH-SY5Y over NTERA2 cells is also supported by the RNAseq analysis, where neural differentiation markers were much higher in the former. Altogether, these results suggest that *MDL1AS* reduction favors neural differentiation in SH-SY5Y cells (more differentiated), but not in NTERA2 (more undifferentiated). The differentiation phenotype induced by *MDL1AS* downregulation is again, cell type dependent (Figure 7).

Another interesting finding is that *DCX*, one of these neural markers overexpressed under *MDL1AS* downregulation in SH-SY5Y cells, diminishes radioresistance in glioma cells, interferes with cell cycle turnover, and increases radiation-induced apoptosis [76]. Furthermore, studies in rat forebrain show that *DCX* is important in neuroblast maturation in response to radiation [77].

Collectively, our results show that *MDL1AS* participates in mitochondrial metabolism, cell differentiation and resistance to radiation. The underlying mechanisms need to be studied in more depth since, apparently, these effects are context dependent. Our main hypothesis is that the degree of differentiation of the cell determines the effects of *MDL1AS*, but further mechanistic studies need to be performed to confirm causality. For future experiments, the use of other cell models, and even primary cells, might provide supportive information. The effects of *MDL1AS* on a cell line and its direct differentiated or radioresistant counterpart [78] might also provide direct information that would be very informative.

In summary, this study demonstrates that *MDL1AS* acts in a context-dependent manner on radiation resistance, tumor metabolism, and cell differentiation. This is the first study to associate the expression of *MDL1AS* with differentiation and radioresistance. Although more research is needed, these results might have a clinical impact in the future. On the one hand, *MDL1AS,* which has already been shown to provide relevant biomarker information [31], might represent a future predictive biomarker about the response of different patients to specific treatments, including radiotherapy. On the other hand, *MDL1AS* could be used as a therapeutic target, since inhibiting *MDL1AS* might sensitize some tumor cells to radiation therapy.

## 5. Conclusions

The results show that *MDL1AS* regulates cancer metabolic preference, radiosensitivity, and differentiation, and this regulation might be dependent on the level of stemness of the cells. Although the mechanistic underpinnings require further study, the paper shows that *MDL1AS* might be considered as a prognostic biomarker and therapeutic target, for example, which may help predict differential responses to radiotherapy.

## Figures and Tables

**Table 1:** Sequence of the DsiRNAs used to downregulate *MDL1AS* expression.

Name	Sequence 5′ → 3′	Strand
DsiRNA2	GUACUACAGGUGGUCAAGUAUUUAT	+
	AUAAAUACUUGACCACCUGUAGUACAU	−
DsiRNA3	GUCGGAUACAGUUCACUUUAGCUAC	+
	GUAGCUAAAGUGAACUGUAUCCGACAU	−
DsiRNA4	GACAUUCAAUUGUUAUUAUUAUGTC	+
	GACAUAAUAAUAACAAUUGAAUGUCUG	−

**Table 2:** Sequence of the primers used for qRT-PCR.

Target	Forward 5′ → 3′	Reverse 5′ → 3′
*MDL1AS*	ACATTACTGCCAGCCACCAT	TGCTTGTAAGCATGGGGAGG
*MDL1AS*	GTCCCTTGACCACCATCCTC	GGGGAACGTGTGGGCTATTT
*ND1*	CCTCCTACTCCTCATTGTACCC	CAGCGAAGGGTTGTAGTAGC
*GAPDH*	AAATCCCATCACCATCTTCC	GACTCCACGACGTACTCAGC

**Table 3:** Antibodies used for Western blotting.

Blotting	Target	Species	Ab Type	Dilution	Reference	RRID
1st	Pai-1	Mouse	Monoclonal	1:1000	Santa Cruz (sc5297)	AB_628154
	Lamin B1	Rabbit	Polyclonal	1:1000	Abclonal (A1910)	AB_2862592
	GAPDH	Mouse	Monoclonal	1:10,000	Abcam (ab8245)	AB_2107448
2nd	Mouse IgG	Donkey	Polyclonal	1:30,000	Jackson Immunoresearch (715-035-151)	AB_2340771
	Rabbit IgG	Goat	Polyclonal	1:10,000	Cell Signaling (7074)	AB_2099233

**Table 4:** Characterization of *MDL1AS* gene downregulation by different DsiRNAs after 48 h (n = 2). The average percentage of reduction against the control is shown.

	NTERA2	SH-SY5Y
**DsiRNA2**	19.72%	69.27%
**DsiRNA3**	50.87%	67.63%
**DsiRNA4**	24.05%	52.46%

## Data Availability

The BAM archives for all sequenced samples are available in the NCBI database under the project accession number PRJNA1357341.

## Supplementary Materials

The following supporting information can be downloaded at: https://www.mdpi.com/article/10.3390/cancers18060928/s1. Figure S1: LPF validation for *MDL1AS* downregulation with siRNAnc, Figure S2: Viability measurements in response to radiation with other DsiRNAs in NTERA2 and SH-SY5Y, Figure S3: whole Western blot membranes, Figure S4: metabolic assay with other DsiRNAs in NTERA2, Figure S5: metabolic assay with other DsiRNAs in SH-SY5Y, Table S1: CSCs markers information, Table S2: neuron-related markers information, Table S3: CSCs and neuron-related genes expression in our samples.

## Abbreviations

The following abbreviations are used in this manuscript:

lncRNA	Long non-coding RNA
*MDL1AS*	*Mitochondrial D-loop 1 antisense lncRNA*
DDR	DNA damage response
CSC	Cancer stem cell
SASP	Senescence-associated secretory phenotype
OXPHOS	Oxidative phosphorylation
FBS	Fetal bovine serum
DsiRNA	Dicer-substrate small interfering RNA
LPF	Lipofectamine
OCR	Oxygen consumption rate
RA	Retinoic acid
PCA	Principal component analysis
DEG	Differentially expressed gene
FC	Fold change
Rot	Rotenone
AA	Antimycin A
