# Supplementary material for: Downregulation of the Long Non-Coding RNA MDL1AS Alters Metabolism, Differentiation, and Radiosensitivity in NTERA2 and SH-SY5Y Cells
Source: Cancers (Basel). 2026 Mar 12;18(6):928. doi: 10.3390/cancers18060928 (PMC13024438; doi:10.3390/cancers18060928)
Supplement: Supplementary file 1 [file cancers-18-00928-s001.zip › cancers-4195801-supplementary.pdf]

cancers

MDPI

Downregulation of the long non-coding RNA *MDL1AS* alters metabolism, differentiation and radiosensitivity in NTERA2 and SH-SY5Y cells

Adrián Casas-Benito [1], Pablo Garrido [1] and Alfredo Martínez [1,*]

[1] Angiogenesis Group , Oncology Area, Center for Biomedical Research of La Rioja (CIBIR), 26006 Logroño, Spain: acasas@riojasalud.es (A.C.-B.); pgarridor@riojasalud.es (P.G.)
* Correspondence: Alfredo Martínez, amartinezr@riojasalud.es

1. Supplementary materials

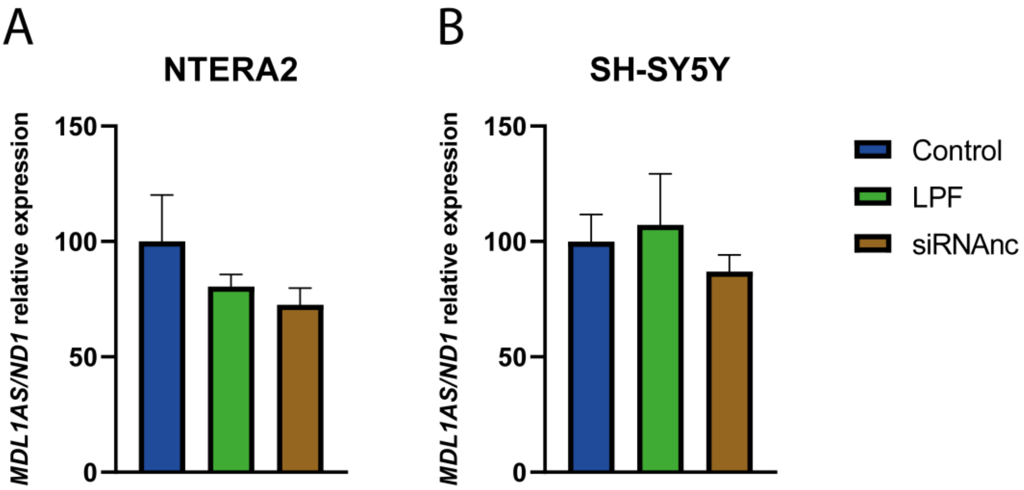

Figure S1. *MDL1AS* expression levels measured by qPCR in medium-only controls (blue), LPF-controls (green) and siRNAnc-treated (brown) cells in both cell lines: NTERA2 **(A)** and SH-SY5Y **(B)**. *MDL1AS* expression was quantified with the second pair of *MDL1AS* primers and normalized to *ND1* expression. All values were expressed relative to the medium-only control. Statistical analysis was performed using one-way ANOVA followed by Tukey's multiple comparison test. No significant differences were observed.

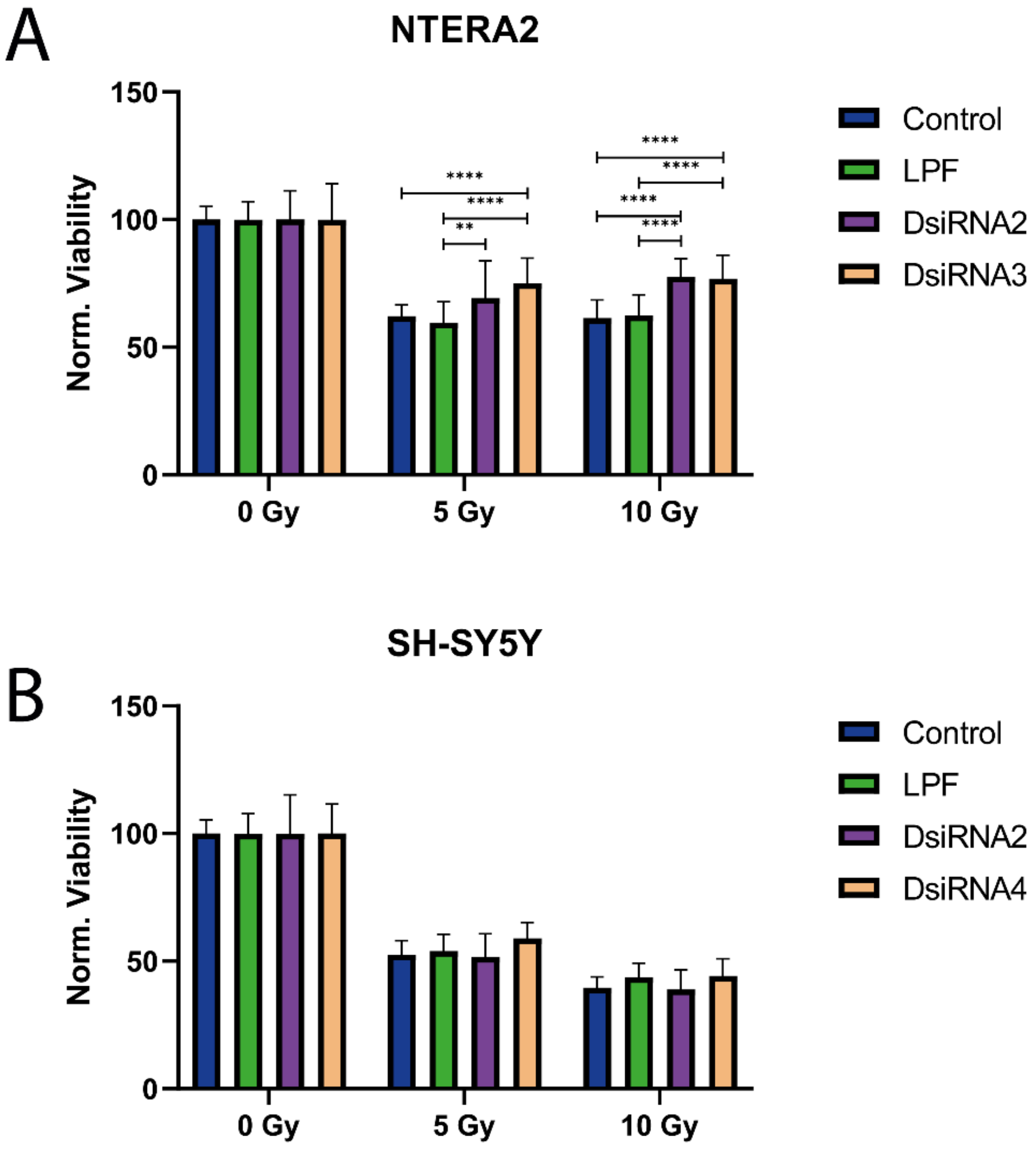

Figure S2. Cell viability of the different groups: medium-only control (blue), LPF control (green), DsiRNA2-treated (purple) and DsiRNA3-treated (orange) in NTERA2 **(A)** or DsiRNA4-treated (orange) in SH-SY5Y **(B)** cells in response to different radiation doses. Samples were collected 24 hours after the exposure to 0, 5, and 10 Gy of radiation. Cell viability was estimated by the MTS protocol. Each of the irradiated conditions were normalized to the non-irradiated group with the same pre-treatment. Statistical analysis was performed using two-way ANOVA followed by Tukey's multiple comparisons test. **: $p < 0.01$; ****: $p < 0.0001$.

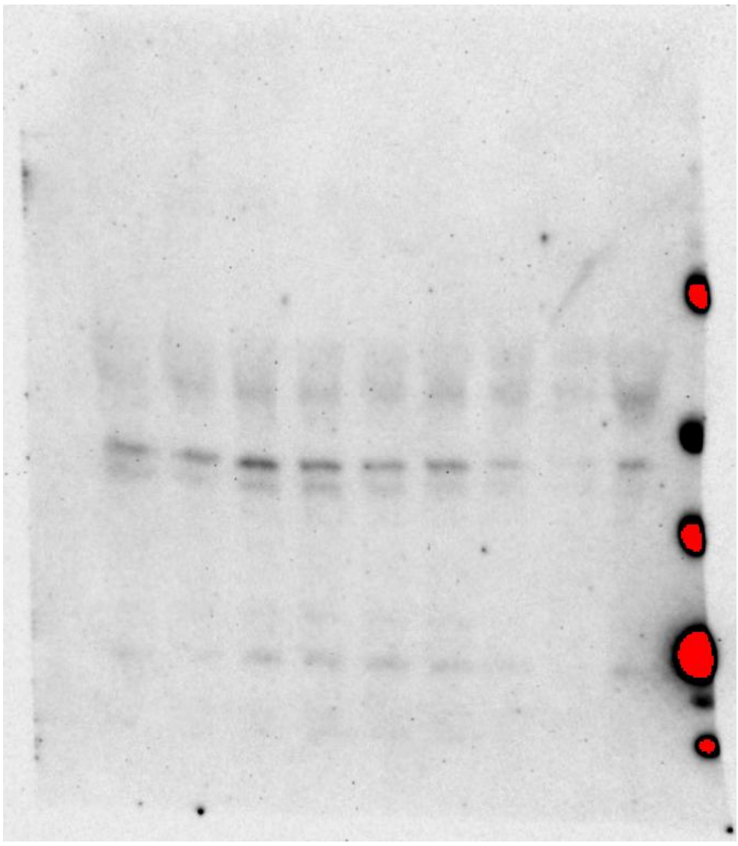

Figure S3A. Western Blot PAI-1 NTERA2. Whole Western Blot membrane from protein supernatant detecting PAI-1 in NTERA2 cells. The selected bands appear in Figure 4.

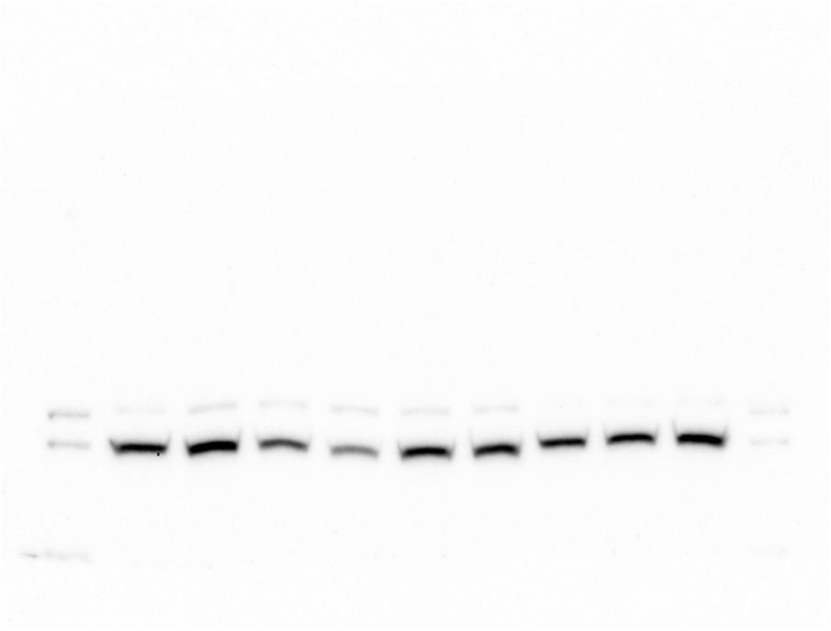

Figure S3B. Western Blot Lamin B1 NTERA2. Whole Western Blot membrane from protein extract detecting Lamin B1 in NTERA2 cells. The selected bands appear in Figure 4.

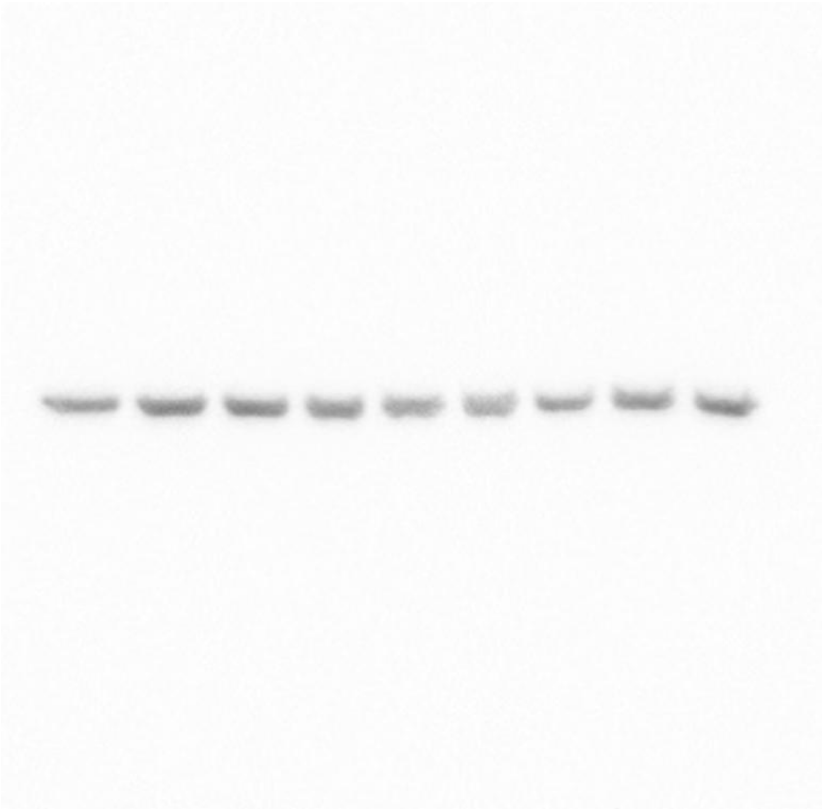

Figure S3C. Western Blot GAPDH NTERA2. Whole Western Blot membrane from protein extract detecting GAPDH in NTERA2 cells. The selected bands appear in Figure 4.

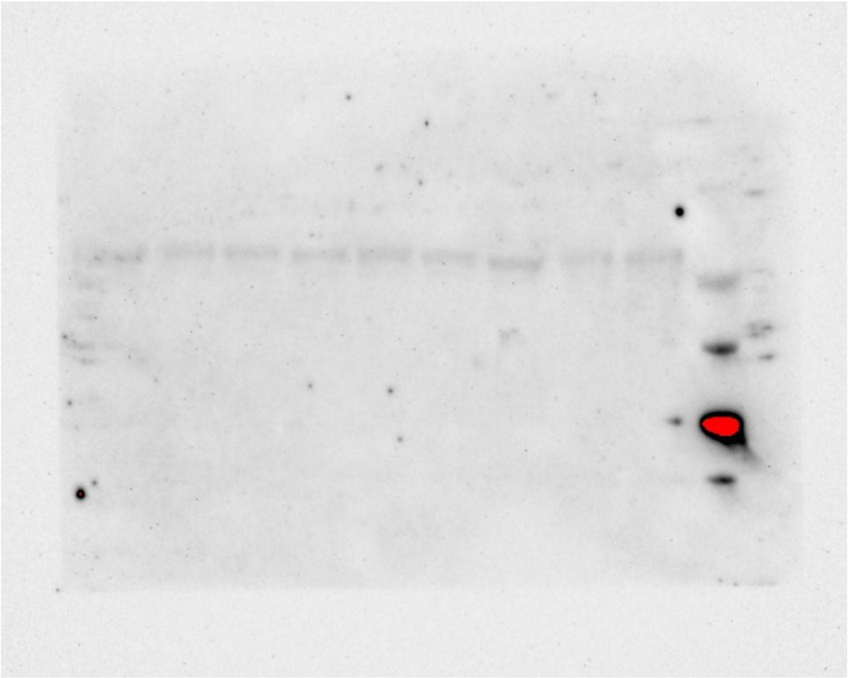

Figure S3D. Western Blot PAI-1 SH-SY5Y. Whole Western Blot membrane from protein supernatant detecting PAI-1 in SH-SY5Y cells. The selected bands appear in Figure 4.

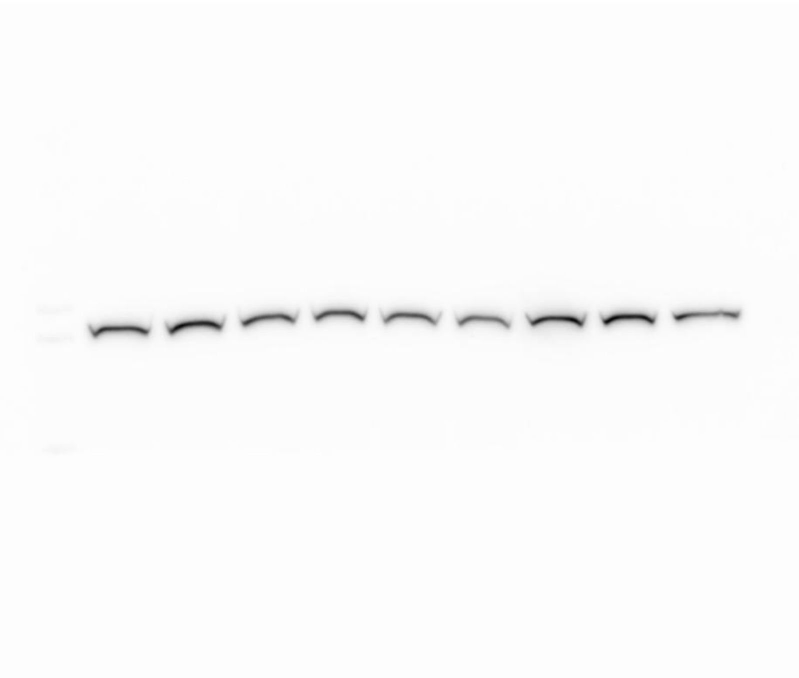

Figure S3E. Western Blot Lamin B1 SH-SY5Y. Whole Western Blot membrane from protein extract detecting Lamin B1 in SH-SY5Y cells. The selected bands appear in Figure 4.

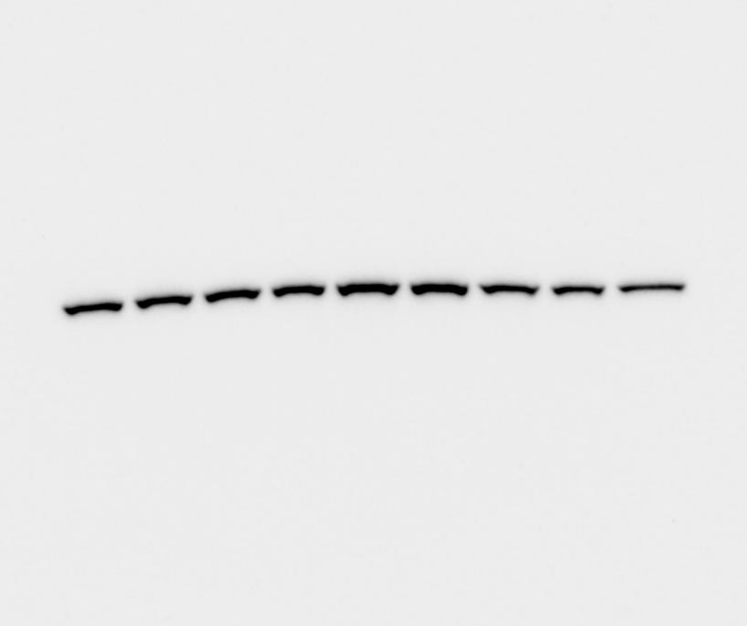

Figure S3F. Western Blot GAPDH SH-SY5Y. Whole Western Blot membrane from protein extract detecting GAPDH in SH-SY5Y cells. The selected bands appear in Figure 4.

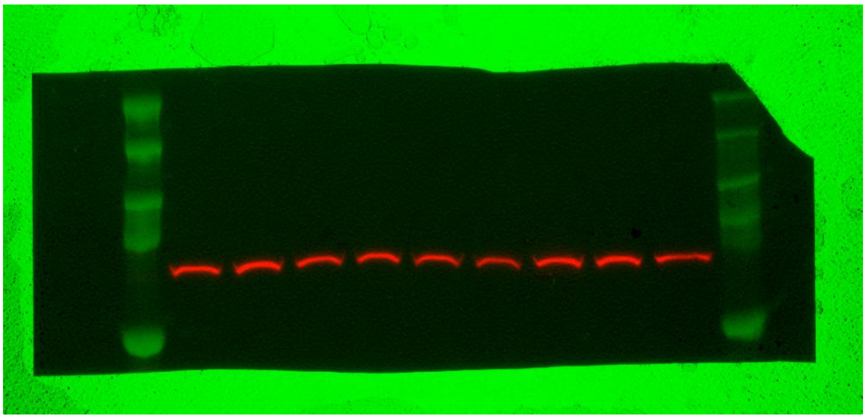

Figure S3G. Western Blot colorimetric molecular weight marker (upper part of the SH-SY5Y membrane).

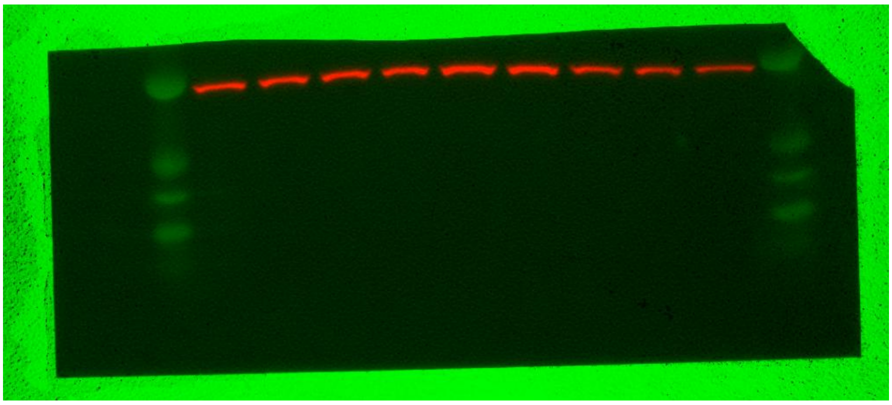

Figure S3H. Western Blot colorimetric molecular weight marker (inferior part of the SH-SY5Y membrane).

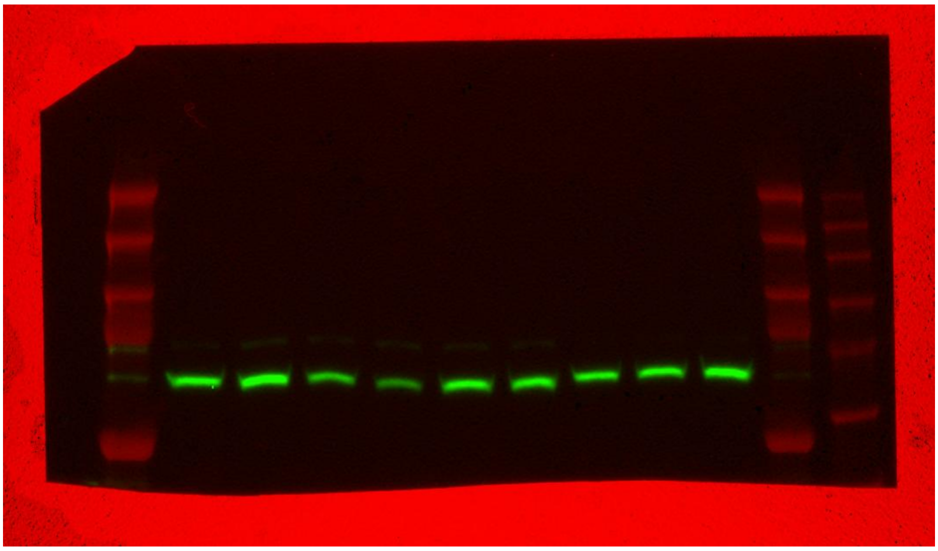

Figure S3I. Western Blot colorimetric molecular weight marker (upper part of the NTERA2 membrane).

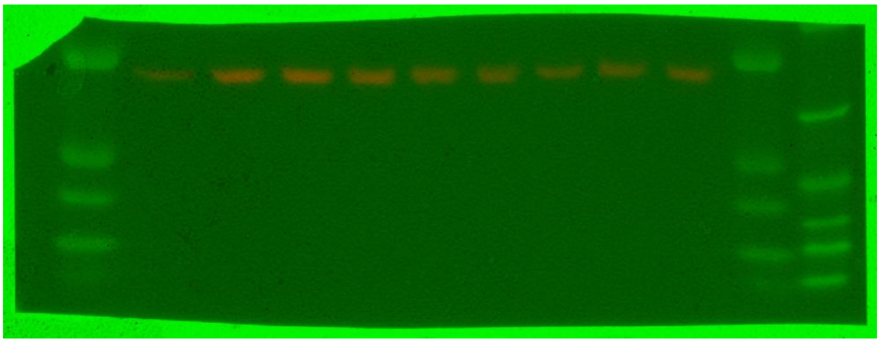

Figure S3J. Western Blot colorimetric molecular weight marker (inferior part of the NTERA2 membrane).

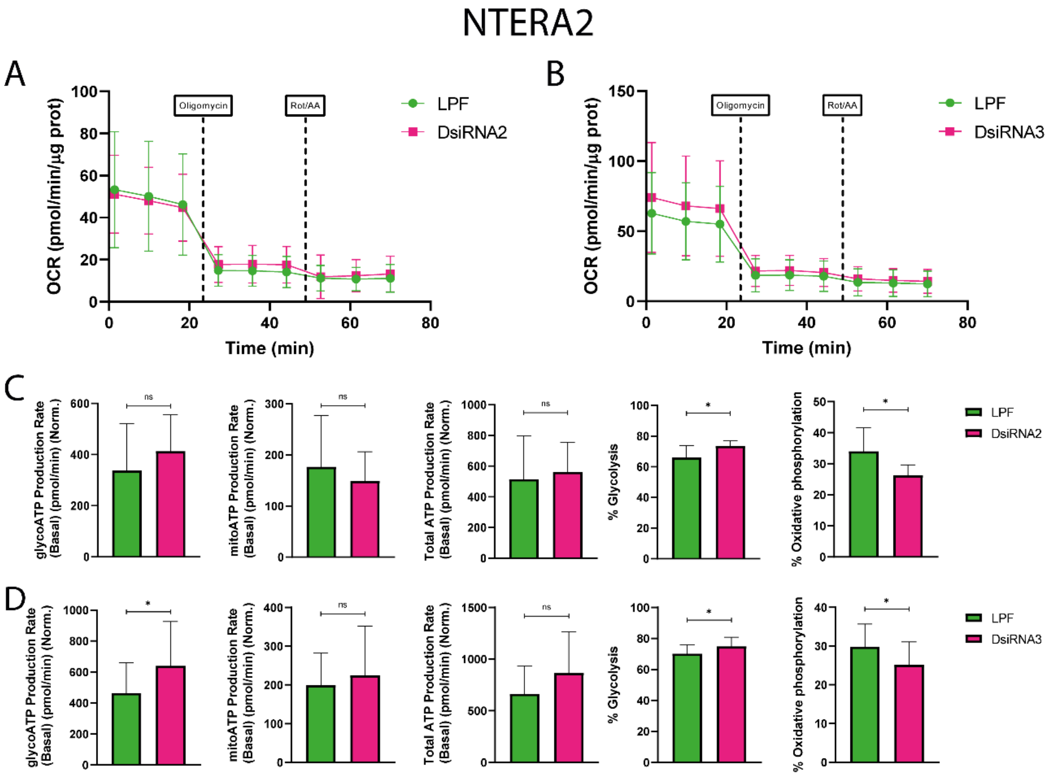

Figure S4. (A and B) Representative graphs showing the OCR in an ATP Rate assay of LPF- (green) and DsiRNA2 (A) or DsiRNA3-treated (B) (red) NTERA2 cells. Oligomycin (at 24 min.) and Rotenone / Antimycin A (at 48 min.) injections are indicated. **(C and D)** Different metabolic parameters derived from the interpretation of the OCR and ECAR of the ATP Rate assay for NTERA2 cells treatment with DsiRNA2 (C) or DsiRNA3 cells (D). The parameters represented are: glycoATP, mitoATP and total ATP production rate and the percentage of glycolysis and Oxidative phosphorylation. Statistical analysis was performed using Unpaired *t*-test. ns: non-significant; *: $p < 0.05$.

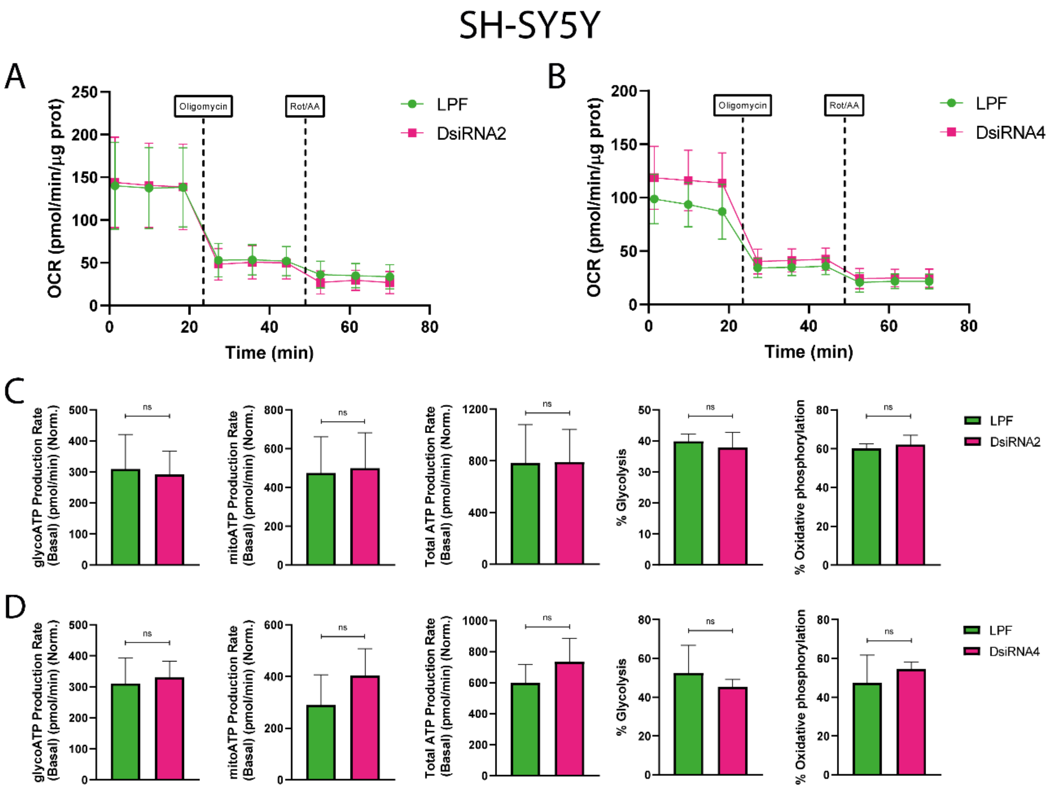

Figure S5. (A and B) Representative graphs showing the OCR in an ATP Rate assay of LPF- (green) and DsiRNA2 (A) or DsiRNA4-treated (B) (red) SH-SY5Y cells. Oligomycin (at 24 min.) and Rotenone / Antimycin A (at 48 min.) injections are indicated. **(C and D)** Different metabolic parameters derived from the interpretation of the OCR and ECAR of the ATP Rate assay for SH-SY5Y cells treatment with DsiRNA2 (C) or DsiRNA4 cells (D). The parameters represented are: glycoATP, mitoATP and total ATP production rate and the percentage of glycolysis and Oxidative phosphorylation. Statistical analysis was performed using Unpaired *t*-test. ns: non-significant.

Table S1. List of CSCs marker genes with the full name and a reference supporting the evidence as a CSCs marker.

CSC marker	Full name	Reference
HES1	*Hes family bHLH transcription factor 1*	10.1016/j.aanat.2021.151848
NES	*Nestin*	10.1111/cas.12691
POU5F1 (OCT4)	*POU class 5 homeobox 1 (octamer-binding transcription factor 4)*	10.1016/j.bbadis.2019.03.005
NANOG	*Nanog homeobox*	10.1016/j.gene.2022.146448
KLF4	*Kruppel like factor 4*	10.3389/fimmu.2025.1514780
PODXL	*Podocalyxin like*	10.1038/bjc.2011.295
CD44	*CD44 "molecule"*	10.1186/s12935-025-03748-4
ALDH1A1	*Aldehyde dehydrogenase 1 family member A1*	10.18632/oncotarget.6920
PROM1 (CD133)	*Prominin 1*	10.1007/s11888-011-0106-1
CD24	*CD24 molecule*	10.1016/j.tranon.2020.100819

Table S2. List of neuron-related markers with the full name and a reference supporting the evidence as a neuron-related marker.

Neuron-related marker	Full name	Reference
DCX	*Doublecortin*	10.3389/fnmol.2017.00199
MAP1B	*Microtubule Associated Protein 1B*	10.1016/j.mcn.2016.01.001
MAP2	*Microtubule Associated Protein 2*	10.1016/j.etap.2025.104904
SYP	*Synaptophysin*	10.1007/s10571-025-01623-4
ASCL1	*Achaete-scute family bHLH transcription factor 1*	10.3390/cells9102230
GAP43	*Growth Associated Protein 43*	10.1016/j.neulet.2026.138520

Table S3. List of genes associated to CSCs (undifferentiation) and to neurogenesis differentiation. The normalized mean for every control and DsiRNA group is represented and the *p* adjusted value (padj) and the log_2FC are indicated. In the last two columns, padj and log_2FC for the comparison between NTERA2 and SH-SY5Y untreated controls are shown. Statistical significant differences are represented in bold case. The positive fold changes are shown in green and the negative ones in red.

	NTERA2				SH-SY5Y				Cell lines control comparison	
Gene	Control	DsiRNA	padj	log2FC	Control	DsiRNA	padj	log2FC	padj	log2FC
CSC (undifferentiation) - associated genes										
HES1	1096	1059	5,86E-01	-0.042	58	49	6,19E-01	-0.058	**3,6999E-211**	**-4.366**
NES	11328	11905	**4,22E-02**	**0.069**	6762	6711	8,74E-01	-0.01	**5,20E-110**	**-0.885**
OCT4	16	10	3,38E-01	-0.114	0	0	NA	0	**1,22537E-08**	**-6.139**
NANOG	1695	1330	**2,06E-05**	**-0.335**	0	0	NA	0	**6,60E-15**	**-12.532**
KLF4	197	361	3,38E-01	0.823	0	2	NA	0.02	**2,21E-03**	**-9.612**
PODXL	56486	52863	**2,79E-04**	**-0.093**	1616	1645	8,21E-01	0.021	**5,00E-04**	**-5.27**
CD44	239	315	**5,08E-03**	**0.304**	21	24	NA	0.026	**1,57087E-39**	**-3.652**
ALDH1A1	2	4	NA	0.035	0	0	NA	0	**3,67E-02**	**-2.105**
CD133	1877	1945	4,23E-01	0.048	349	215	**9,07E-08**	**-0.557**	**1,1165E-240**	**-2.563**
CD24	16225	10212	**5,17E-145**	**-0.666**	21	24	NA	0.026	**7,04E-235**	**-1.115**
Neurogenesis differentiation - associated genes										
DCX	214	181	1,96E-01	-0.146	2235	4442	**2,34E-86**	**0.982**	**2,48E-189**	**3.239**
MAP1B	6184	6102	6,58E-01	-0.019	14473	16621	**3,55E-09**	**0.185**	**1,02E-21**	**1.076**
MAP2	1860	1247	**2,39E-16**	**-0.564**	6916	8272	**9,60E-03**	**0.248**	**1,94E-227**	**1.749**
SYP	268	262	8,81E-01	-0.02	1661	1913	**1,51E-02**	**0.168**	**4,94E-167**	**2.48**
ASCL1	11	7	NA	-0.049	2673	2474	1,94E-01	-0.089	**3,19E-165**	**7.91**
GAP43	919	800	**2,23E-02**	**-0.162**	3041	2984	7,48E-01	-0.023	**3,73E-170**	**1.588**
